# Combining Higher-Order Logic with Set Theory Formalizations

**DOI:** 10.1007/s10817-023-09663-5

**Published:** 2023-05-25

**Authors:** Cezary Kaliszyk, Karol Pąk

**Affiliations:** 1 Department of Computer Science, University of Innsbruck, Innsbruck, Austria; 2 INDRC, International Neurodegenerative Disorders Research Center, Prague, Czech Republic; 3 Institute of Computer Science, University of Białystok, Białystok, Poland

**Keywords:** Higher-order logic, Set theory, Transport

## Abstract

The Isabelle Higher-order Tarski–Grothendieck object logic includes in its foundations both higher-order logic and set theory, which allows importing the libraries of Isabelle/HOL and Isabelle/Mizar. The two libraries, however, define all the basic concepts independently, which means that the results in the two are disconnected. In this paper, we align significant parts of these two libraries, by defining isomorphisms between their concepts, including the real numbers and algebraic structures. The isomorphisms allow us to transport theorems between the foundations and use the results from the libraries simultaneously.

## Introduction

Among the various foundations for formal proofs, set theory on top of higher-order logic has been tried a number of times in systems such as HOLZF [42], ProofPeer [43], Egal [10], and Isabelle/Mizar [28]. This foundation is attractive for formalization, as it offers a natural mathematical foundation combined with the automation present in HOL.

The formal proof libraries of Isabelle/HOL [55] and that of Mizar [4, 16] are among the largest proof libraries in existence today. Indeed, the HOL library together with the Archive of Formal Proofs consist of more than 100,000 theorems [6], while the Mizar Mathematical Library (MML) contains 59,000 theorems. Furthermore, the results contained in the libraries are incomparable: Almost all of the Mizar library concerns itself with mathematics, while the majority of the Isabelle/AFP library are results closer to computer science [6]. For example, the Mizar library includes results about lattice theory [9], topology, and manifolds [46] not present in the Isabelle library, while the Isabelle library has many results related to algorithms not in the MML [13, 36, 37].

In our previous work [7], we have presented a model of higher-order Tarski–Grothendieck, which justifies the use of higher-order logic formalizations with set theory-based ones simultaneously. This model will allow us to combine the results present in these two major Isabelle libraries. We will specify isomorphisms between various basic types present in the libraries, such as functions and lists, leading to isomorphisms between various number structures including the real numbers, and algebraic structures. The last requires mappings between extensible soft record types and Isabelle type classes [24].

We will use the isomorphisms to transport proved theorem including the theorems of Lagrange, Bertrand, cases of Fermat’s last theorem and the Intermediate Value Theorem. We will also merge the formalizations of groups and rings in the two libraries.

This paper is an extended version of our paper presented at ITP 2019 [7]. In particular the new content presented is as follows:

- we specify the alignments between many more complex types in the two proof libraries including the rationals and the real numbers;
- we transfer more advanced theorems between the two foundations, including the intermediate value theorem in the merged HOL-Set theory library, together with a large set of theorems that connect Dedekind cuts with Cauchy sequences; and
- we complete the model of higher-order Tarski–Grothendieck presented in our previous work [7], by justifying that the Grothendieck-style axioms are equivalent to the Tarski style (for example used in the Mizar Mathematical Library), formalizing the relationship between them in Isabelle.

The rest of the paper is structured as follows. In Sect. 2, we introduce the Isabelle HOTG foundations, which will be the basis for all the work, we describe the various axiomatizations of higher-order Tarski–Grothendieck (HOTG) and prove some of them to be equivalent. The basics of the aligned libraries are presented in Sect. 3. The subsequent Sects. 4 and 5, 6 discuss our isomorphisms between the different types concerning functions, numbers, and algebra respectively. Section 7 shows practical examples of theorems we can move using the isomorphisms. Section 8 discusses the Tarski–Grothendieck equivalence proofs. Finally, Sect. 9 discusses the related work on combining foundations and Sect. 10 presents the existing automated transfer methods in higher-order logic and discusses the limitations of the current work in this respect.

## Isabelle and Isabelle/Mizar

The Isabelle logical framework’s meta-logic *Pure* is a variant of simple type theory with shallow polymorphism. The framework provides functionality that makes it convenient to define object logics, namely allowing easily defining their types, objects, and inference rules as well as their notations. Isabelle/HOL is today the most developed Isabelle object logic. Further Isabelle object logics [48] include constructive type theory or untyped set theory [49].

As Isabelle/HOL is relatively well known and documented, we assume that the reader is familiar with the HOL foundations, Isabelle’s basic commands (such as *definition* and *theorem*) and the basic Isabelle objects (numbers and lists). For details, we refer the reader to the Isabelle Manual [54].

The details of Isabelle/Mizar’s design and implementation have been presented previously [28], therefore, we present only the main commands needed for understanding the current paper. Isabelle/Mizar can be loaded on top of Isabelle/FOL or Isabelle/HOL. It re-uses the type of propositions of the underlying basic logic (o of FOL or bool of HOL) and its basic propositional connectives (negation, conjunction, disjunction, implication), as well as the polymorphic equality present there. However, as the intention of Isabelle/Mizar is to provide a sofly-typed set theory, the universal and existential quantifiers are actually bounded quantifiers that for each quantified object require the type over which it ranges (e.g., $\forall$ *x being Nat. ...*). These propositional and predicate quantifiers together with quality are sufficient for representing firest-order logic with quality and to represent Jaśkowski [26] style natural deduction proofs present in Mizar.

To introduce the soft type system, a meta logic type of soft-types ty is declared together with the an infix operator is that corresponds to the element satisfying the predicate associated with a type. Types can be combined with an intersection operator (e.g., x is even | number) and can be negated (e.g., y is non-negative) with natural semantics to these operations. The meta-logic abstractions can be used to parametrize the types by other types or even by terms (e.g., A is m,n-matrix corresponds to m-by-n matrices). To improve automation, the user can prove properties of types, including *inhabited* and *sethood*. The first one is useful for eliminating quantifiers, whereas the latter is useful for forming compregension operators. Finally, a choice operator (denoted the on the level of types allows for getting a term of a given type). For example, given the type of sets, that is intersected with empty, it is possible to define the empty set as the empty | set.

The Isabelle/Mizar object logic subsequently introduces the axioms of set theory, specifically, the Tarski–Grothendieck axioms. In particular, the Fraenkel axiom is sufficient to construct set comprehensions written as {F(x)where x be Element-of X: P(x)} (called *Fraenkel terms*) for a given set *X*, function *F* and predicate *P*. In the Mizar language, it is not always possible to define such a functor for arbitrary *X*, *F*, *P*, to avoid inconsistency (variants of Russell’s paradox), however, with the help of sethood safe comprehension terms can be interpreted. In Isabelle/Mizar the semantics of comprehension are defined with sethood as a precondition, which means that the property is only valid for terms for which sethood has been proved. This completes the axiomatic part of the object logic, and subsequent parts are introduced as definitional extensions. In particular, the possibility for users to define all kinds types and objects, as well as syntax that allows an easier interaction with softly-typed set theory will be added in this way.

Isabelle/Mizar allows four kinds of user-level definitions corresponding to the same four kinds of user-level definitions in Mizar [16]. Defining predicates is not different from the usual Isabelle definitions. We present the definition of a set theoretic functor by the example of the set theoretic union of two sets^1^: 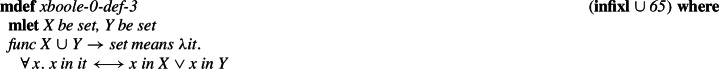 The **mdef** command starts with the handle used to refer to the definition, followed by an optional notation (union denoted by infix $\cup$), a typing environment in which the definition is made (**mlet**) and then the actual defined operator is given after the keyword *func*. The return type is given after the keyword  . A definition by *means* is supposed to correspond to a concept where the *it* has the desired property. The user needs to show the existence and the uniqueness as proof obligations. When the user completes these proofs, the Isabelle/Mizar definition package introduces the identifier together with the theorems corresponding to the property of the object and its type for further use. Functors can also be defined by *equals* where the term is given directly in a given environment and with a given return type of the defined term. There, the obligation is to show that the result has the return type.

Type definitions are similar. In order to make type inference and checking automatable, types are divided into modes (more primitive types that are known to be inhabited) and attributes (the types that are used to restrict other types with intersection). Consider for example the definition of the type of a finite sequences over the type *D* (which are the set-theoretic equivalents of polymorphic lists used are often used in formal proofs): 
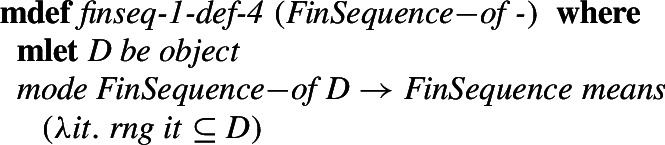
 Again **mlet** introduces an environment (these are preconditions for the definitional theorems but can be used in the proofs) and the definition can describe the desired properties that all objects of the defined type must have. After the proof obligation (non-emptiness) is proved, definitional theorems are derived and given to the user. The already mentioned attributes are also similar. They restrict a given type to a subtype. An example type introduced with the help of an attribute is the type of relations. First, the attribute *Relation_like* is introduced, which can be later used to define the type of relations as just an abbreviation, as follows. 
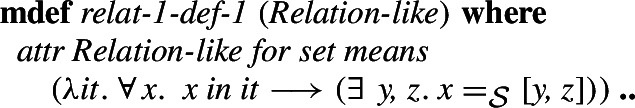
 This approach allows for all definitions and operations defined for a *Relation* to also immediately be available for a *Function*, which is defined as a type restriction using the attribute *Function_like*. The type *FinSequence* is similarly defined by the attribute *FinSequence_like* as follows: 
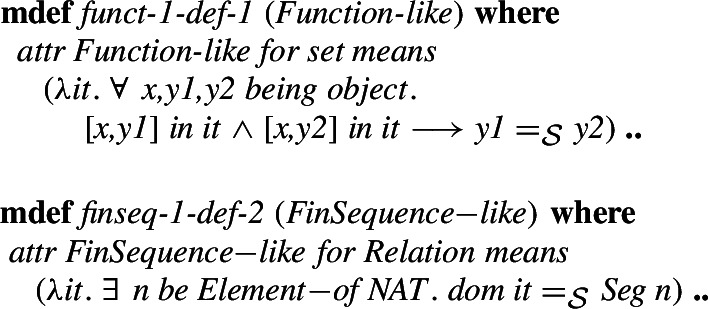

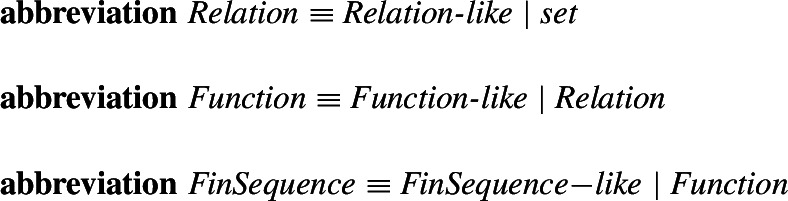
 Finally, Isabelle/Mizar introduces the **mtheorem** command, that is similar to the standard theorem command, but additionally allows the introduction of soft-type assumptions with the **mlet** keyword and hiding these from the user as long as the automated type inference can handle these. Additionally to imitate the Mizar automation the **mby** proof method has been included, that combines type inference with Isabelle’s auto proof method.

Parallel to the system development, the Mizar community puts a significant effort into building the Mizar Mathematical Library (MML) [4]. Parts of the MML library (including numbers or parts of algebra) have been translated to Isabelle/Mizar [29] and are being used in the current paper.

## Proof Integration

The Isabelle higher-order Tarski–Grothendieck foundations allow the import of results proved in higher-order logic and in set theory. This is possible both theoretically (we have previously presented a model that supports the combined foundation [7] and discussed its adequacy more in Sect. 8) and practically, that is the Isabelle logical framework allows us to import various results from the two libraries of Isabelle/HOL and Isabelle/Mizar in the same environment. Note, however, that the imported developments are initially disconnected. In this and the next sections, we will define transfer methods between these results. These will allow us to use theorems proved in one of the foundations using the term language of the other.

All the definitions and theorems presented in these sections have been formalized in Isabelle and will be presented close to the Isabelle notation. The Isabelle environment will import both Isabelle/HOL [41] and Isabelle/Mizar [28] object logics along with a number of results formalized in the standard libraries of the two. Isabelle distinguishes between meta-level implication ($\Longrightarrow$) and object-level implication ($\longrightarrow$) and our notation in examples below reflects this distinction. The remaining notations will follow first-order conventions. In particular, the symbols  and  will refer to the HOL and set-theoretic equality operations respectively. Then, *be* is the Mizar infix operator for specifying the type of a set in the Mizar intersection type system [31].

In order to transfer results between the foundations, we will first define bijections between types that are isomorphic. We will next show that these bijections preserve various constants and operators. This will allow us to transfer results using higher-order rewriting, in the style of quotient packages for HOL [23, 34] and the Isabelle transfer package [21]. Note, that we are not able to use these packages directly. We discuss this in Sect. 10.

In the Mizar set theory there are often two ways to express domains of objects. It is already the case for the natural numbers, where it is common to reason both about the type of the natural numbers and the members of the set of natural numbers. This is necessary since the arguments of all operations must be sets, while the reasoning engine allows more advanced reasoning steps for types [4]. We, therefore, define two operators, one that specifies a bijection between a HOL type and a set-theoretic set and one that specified a bijection between a HOL type and a set-theoretic type. The definitions are analogous and we show only the former one here. We will define an isomorphism between a type $\sigma$ and a set $d\in \Lambda _\iota$ to be a pair (*f*, *g*) of functions (at the type theory level) where *f* maps sets to objects of type $\sigma$ and *g* maps objects of type $\sigma$ to sets in such a way that objects of type $\sigma$ (in the type theory) correspond uniquely to elements of *d* (in the set theory).

### Definition 3.1

Let $\sigma$ be a type, $d\in \Lambda _\iota$ be a set and  and  be functions. The predicate  holds whenever all of the following hold:

,
,
.

In Isabelle the definition appears as follows: 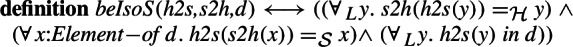 The existence of a bijection does not immediately imply the inhabitation of the type/set. However, as types need to be non-empty in both formalisms, we can derive this result as below. For space reasons we only present the statements, all the theorems are proved in our formalization. 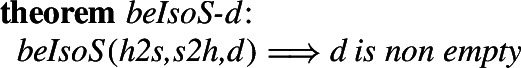


## Integrating Basic Infrastructure: Functions and Lists

We will denote the morphisms from set theory to HOL with the prefix s2h and the inverse ones with the prefix h2s. We will initially give the complete types for readability, omitting them later, where the types are clear. The first type, for which we build an isomorphism, is the type of functions. In order to transfer a function of the type $\alpha \rightarrow \beta$ between set theory and HOL, we will require isomorphisms for the types $\alpha$ and for the type $\beta$.

In order to transfer a set-theoretic function (set of pairs) to HOL, given transfer functions on the range, on the domain, and the function itself, we return the lambda expression, that given a HOL input to the function, transfers it, applies the function to it and transfers it back. The formal definition is as follows. 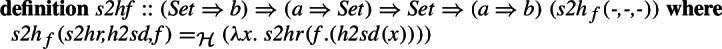 Similarly, to build a set-theoretic function (set of pairs) given a HOL function and the transfer operations, and the domain, we directly build this set: 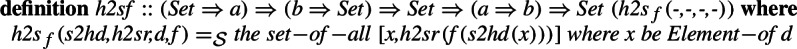 We are then able to directly show that these two functions are inverses of each other on their domains. We also show the existence of an isomorphism, and show that this isomorphism preserves the function application operation: 
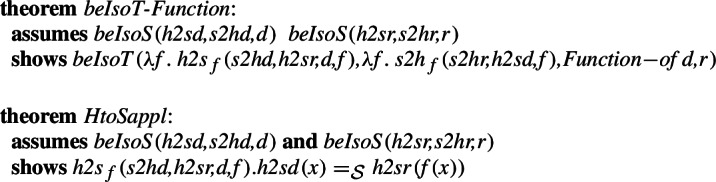
 Isabelle/HOL lists are realized as a polymorphic algebraic datatype, corresponding to functional programming language lists. MML lists (called finite sequences, FinSequence) are functions from an initial segment of the natural numbers. Higher-order lists behave like stacks, with access to the top of the stack, whereas for the set-theoretic ones the natural operations are the restriction or extension of the domain.

To build a bijection between these types, we note that the  operator corresponds to the concatenation of a singleton list and the second argument. Since the list type is polymorphic (in the shallow polymorphism sense used in HOL), in order to build this bijection, we also need to map the actual elements of the list. Therefore the bijection on lists will be parametric on a bijection on elements: 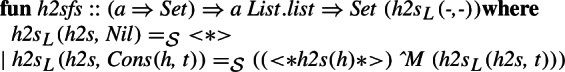


Where  and  represent the Mizar empty sequence and the concatenation of sequences respectively. The converse operation needs to decompose a sequence into its first element  and the remainder of the sequence shifted by one  . We define this operation in Isabelle/Mizar and complete the definition. Isabelle will again require us to show the termination of the function, which can be done by induction on the length of the list/sequence: 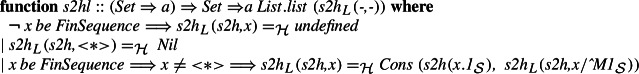 For the transformation introduced above, we can show that if we have a good homomorphism between the elements of the lists, then lists over this type are homomorphic with finite sequences.

We can again show that this homomorphism preserves various basic operations, such as concatenation, the selection of *n*-th element, length, etc. 
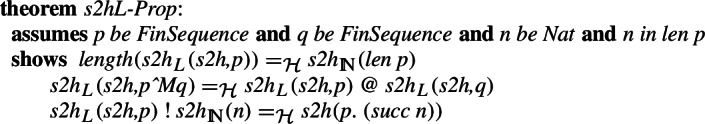


Note, that the sequences in the Mizar library, *FinSequence*, are indexed starting at 1, whereas Isabelle/HOL’s *nth* starts from 0, which justifies the usage of a shift (*succ n*). Furthermore, since Mizar Mathematical Library uses natural numbers in the Peano sense, the expression *n in len p* actually means  . To actually use these in order to move theorems between the libraries we show how the morphisms interact with the operations. For example, for reverse these are: 
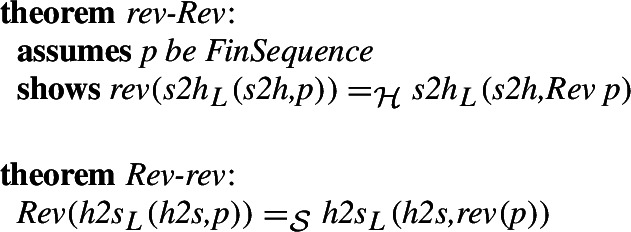


Moving a polymorphic statement from the Isabelle/HOL library to Isabelle/Mizar requires an additional assumption about the existence of an isomorphism on the parametrized type. The usual statement about the length of a reversed list, therefore becomes (of course this simple statement is already available in the Isabelle/Mizar library, and can be used by referring to *finseq_5_def_3*, but its simplicity is good to demonstrate moving polymorphic statements): 
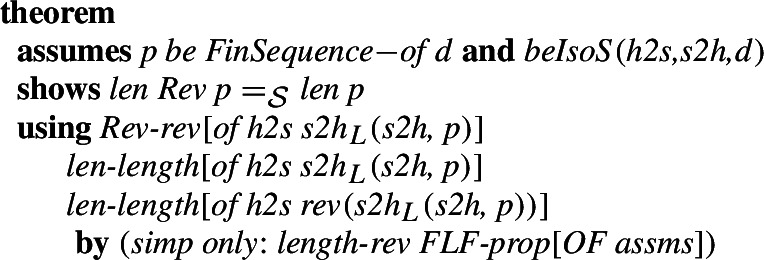


We also show the proof here. It is still straightforward, just like the other proofs of the moved statements given the morphisms, but with polymorphism it no longer follows by higher-order rewriting.

## Numbers

The way numbers are constructed in set-theory based libraries is very different from the majority of the libraries based on HOL or type-theory. In particular, in Isabelle/Mizar subsequently defined number types are extended (in the sense of set-theoretic subset) by new elements. This is as opposed to hard-type-based systems, in which subsequently defined number types are independent and projections or coercions which preserve the functions are necessary. In particular, Isabelle/Mizar’s real numbers are constructed as Dedekind cuts. Note, however, that the cuts corresponding to the rational numbers are replaced by the rational numbers themselves, in order to preserve the inclusion $\mathbb {Q}\subset \mathbb {R}$.

A second, less important, distinction is the fact that in the Mizar library the non-negative types ($\mathbb {N}, \mathbb {Q}^{\ge 0}, \mathbb {R}^{\ge 0}$) are constructed first. After this, the negative reals are built as Kuratowski pairs of the singleton zero and the positive element. Finally, the rationals and integers are subsets of the set of all reals. In particular, the sets $\mathbb {N}, \mathbb {Q}^{\ge 0}, \mathbb {R}^{\ge 0}, \mathbb {R}$ are already constructed with the basic operations on these sets and addition, subtraction, multiplication directly re-use the real operations. The only additional thing to prove is that the types are preserved, so for example the addition of integers returns a real that is also an integer.

**Fig. 1:**
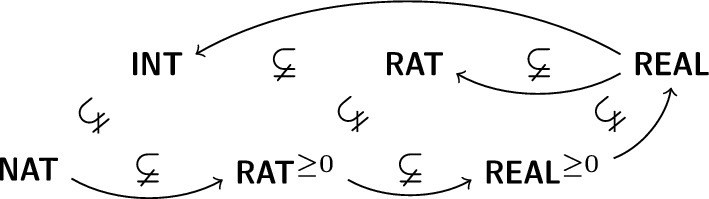
The inclusions between the sets in the Mizar Mathematical Library. The arrows show the construction order between the sets in the MML and our Isabelle set formalization

The inclusions, together with the order of the construction are depicted in Fig. 1. In order to realize this construction in Isabelle/Mizar, we first define the set of the natural numbers, as the smallest limit ordinal. The formal definition is as follows: 
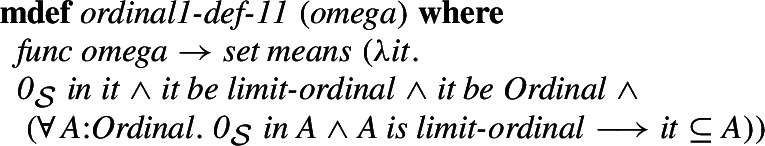


The definition introduces the constant (zero-argument functor) *omega* of the Mizar type *set*, which satisfies the condition specified after the keyword *means*, that is, the defined constant *it* is a limit ordinal with  as a member, and it is the smallest such set (considering set inclusion). As a reminder, the **mdef** command requires the formalization to specify the existence of the constant (proof is only included in the formalization), which is a consequence of the Tarski universe property and its uniqueness.

On the other hand, the Isabelle natural numbers are a subtype of the type of individuals. In order to merge these two different approaches, we specified a functor that preserves zero and the successor. Note that the functor is specified only for the type of the natural numbers which in Isabelle/HOL is implicit, but in the softly-typed set theory needs to be written and checked explicitly. This is the reason for having an undefined case, which as we will see later, still gives an isomorphism.The functor and its inverse are formally defined in Isabelle as follows 
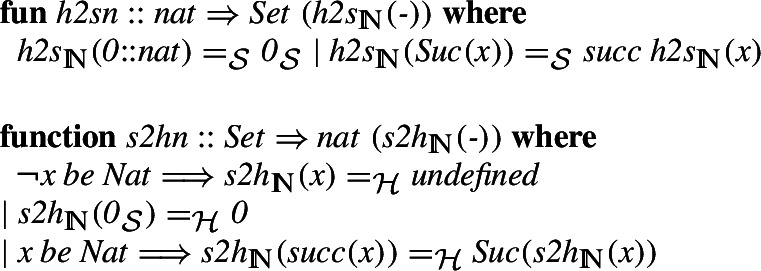


Note that  is defined only on the HOL natural numbers ( ), while  is defined on all sets and its definition is only meaningful for arguments that are of the type  . The soft-type system of Mizar requires us to give this assumption explicitly here, but it can normally be hidden in the contexts where the argument type is restricted appropriately. Isabelle requires us to prove the termination of the definition, which can be done using the proper subset relation defined on natural numbers in the Peano sense.

Using the induction principles for natural numbers present in both libraries, we can show the property  , where  is the set of all  . In particular, it gives a bijection (note the hidden type restriction to sets of type  ). We show also that the functors  ,  preserve all the basic operations. 
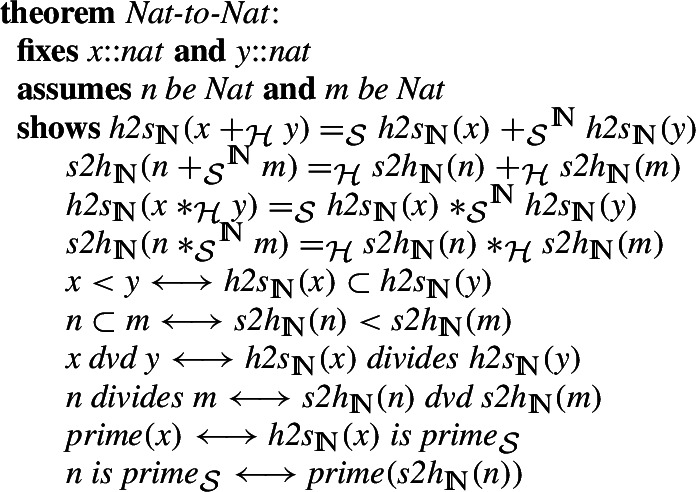


### Isabelle/Mizar Number Hierarchy

After the natural numbers, MML constructs the non-negative rationals as pairs of relatively prime naturals. Additionally, to preserve the set-theoretic inclusion of the set of natural numbers, not only pairs with the denominator zero but also those with denominator one are excluded and the original natural numbers added. We follow the same construction in Isabelle/Mizar. 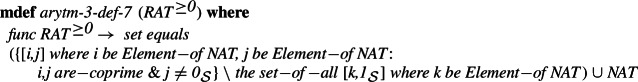


Non-negative real numbers are constructed in a similar way. To the set of non-negative rationals, we add Dedekind cuts corresponding to the positive irrational numbers. A standard definition of Dedekind cuts is used, only restricted to non-negative rationals. We assume that a proper subset  of non-negative rationals is a cut, if it is closed under smaller elements () and for every element in the set  there is a larger element in the set  ( ). Note that  fulfills this condition, however, it is not a proper subset of non-negative rationals. In contrast, in this approach, the empty set is a Dedekind cut, but we do not need to add it in the construction of , since empty corresponds to zero. 
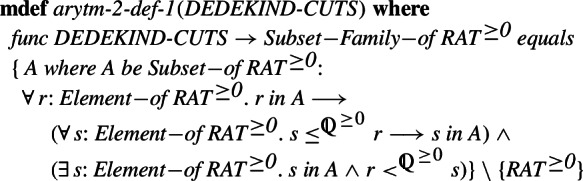


In order to preserve the inclusion between the rationals and reals, again the non-negative real numbers are obtained as a union of the non-negative rationals as defined above and the Dedekind cuts corresponding to the irrational numbers, that is cuts that cannot be realized in the form  where  is rational. 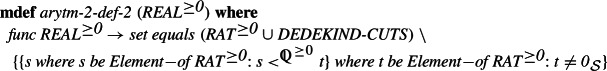


Finally, the complete reals ( ) are constructed by adding the negative real numbers. In the Mizar set theory the negative numbers are represented by the pairs  , where  is a positive real number. For this, we add the pairs corresponding to  , where  is a non-negative real and then remove the pair  to avoid duplicating 0. The sets of rationals and integers are then appropriate subsets of the set  . Of course, it would be possible to build these sets directly, together with their respective arithmetic operations, however, this would require the introduction of different symbols for these operations in the different datatypes. The Isabelle/Mizar formalization only temporarily introduces the operations $\mathbb {Q}^{\ge 0}, \mathbb {R}^{\ge 0}$ which will almost never be used in the library, and the operations for the type $\mathbb {R}$, which will be directly reused for $\mathbb {Z}$ and $\mathbb {Q}$. In particular, this allows using the operations in the context of homomorphisms between integers, rationals, and reals. 
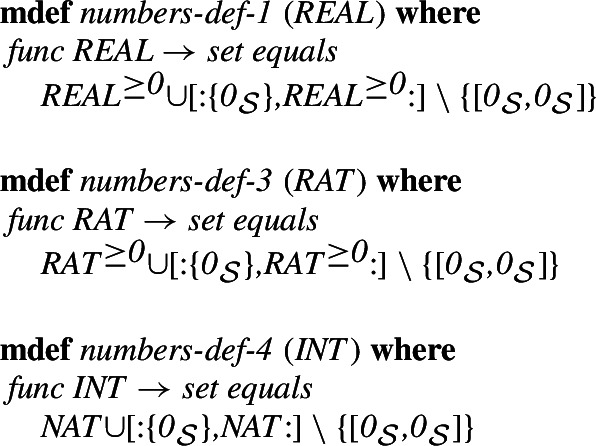


### Integrating Numbers

Given the Isabelle/Mizar number hierarchy specified in the previous section, we can start building bridges between the types. We start with the integers. The set-theoretic definition is again different from the one used in Isabelle/HOL. There, an equivalence relation (equal modulo the difference) is defined on pairs of natural numbers, and the quotient package [34] is used to construct the new type. Still, it is straightforward to define a bijection between the two, using the constructed bijections between natural numbers. We also show that these bijections preserve all the basic operators. 
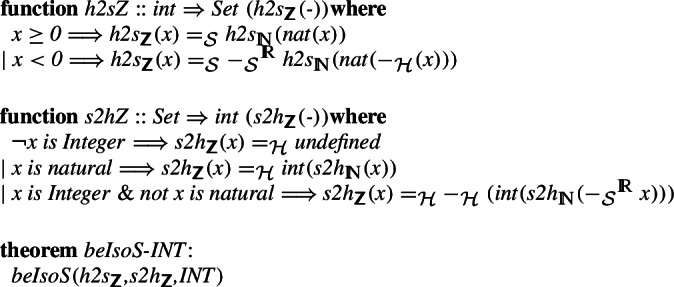


For the rational numbers, we construct the natural bijection  ,  using the bijections between the integers and the unique representation of any rational as an irreducible fraction. We again show that the operations behave well on arbitrary (including reducible) fractions. 
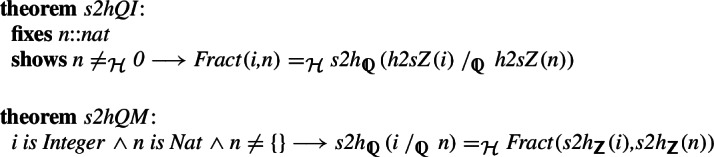


The constructions of the real numbers are significantly different in the two considered proof libraries. Indeed, in Isabelle/HOL reals are quotients of Cauchy sequences whereas the MML one uses Dedekind cuts. More precisely, in the MML, Dedekind cuts are used to construct the irrational, and operations on them are defined on the cuts. To build a homomorphism between the two definitions and to use it for all the operators requires considering cases, namely whether the given argument is a rational number of a cut. The same is true for the results of the operators.

To ease these constructions we first introduce two operators: *DEDEKIND_CUT* which transform a real number to a Dedekind cut, i.e., for positive rationals it associates to the number  the cut  and for irrational numbers, which are already cuts, it is the identity. We also define the inverse operator *GLUE*, which transforms cuts that can be represented in the form  for a rational  , returns  , and is the identity otherwise. 
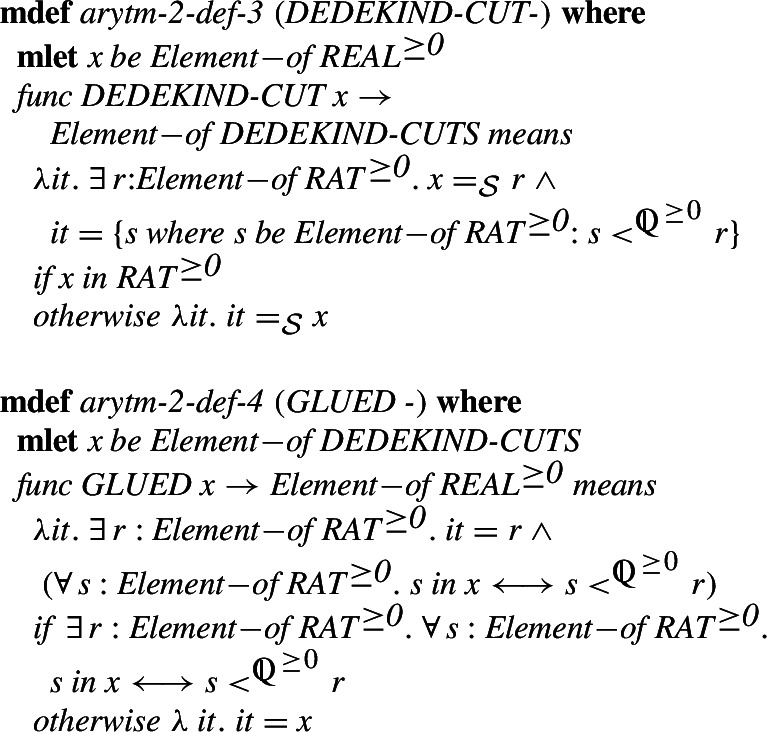


We will now construct the homomorphism between the real number representations. Consider a non-empty Dedekind cut  . We observe, that by multiplying all the elements of  by a positive rational  , we obtain a non-empty Dedekind cut. We denote this cut by  . Next, we denote by  the largest natural number in the set  . Consider the sequence of non-negative rationals . It easily follows that this sequence is non-decreasing and that for every $n \le k$ it is true thatwhich shows that this sequence is a Cauchy sequence.

This allows us to associate any positive real number with a Cauchy sequence of rationals: 
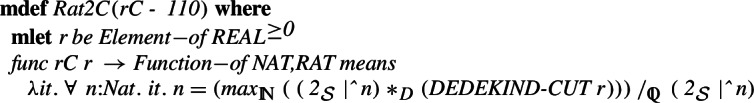


Using the previously defined homomorphisms between the naturals and rationals as well as between the types of functions (Sect. 4 and previous subsections of Sect. 5), we can transform this set-theoretic function to a HOL one. We show that this transformation preserves Cauchy convergence: 
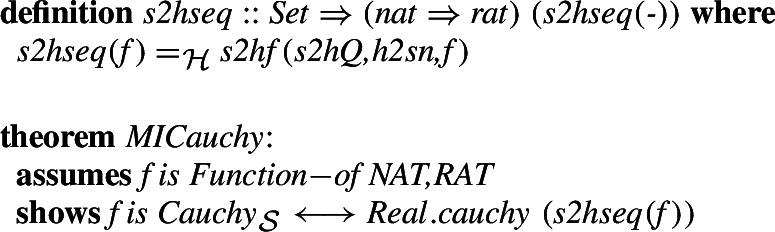


Which allows us to define the final homomorphism that given a set-theoretic real transforms it to a HOL real. 
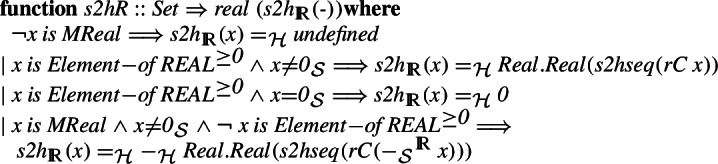


where for non-negative real number  , we use it to produce the sequence of rational numbers  , which are subsequently transformed to a sequence of HOL reals  , and finally we return the abstraction of the Cauchy sequence class to which the sequence belongs. For negative real numbers, we use minus twice, analogously to the integer and rational constructions. 

In order to build the inverse transformation, we will construct the Dedekind cut based on a real number. First, for any real number  , we start with one of the Cauchy sequence  belonging to its equivalence class  . We consider the equivalence of this sequence in set theory:  . This sequence is non-decreasing and has non-negative values if  is non-negative. Additionally, if  is positive, this sequence  is also positive starting from some index. This means that for any positive real  , the sequence  is non-empty (from some position, to be precise when  ) and non-decreasing and its union ( ) is a Dedekind cut. 
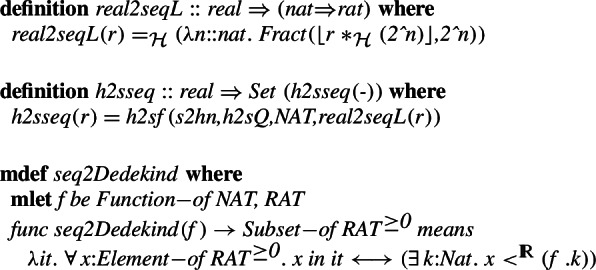


The final transformation that given a HOL real number extracts its Cauchy sequence and transforms it to an Isabelle/Mizar real is: 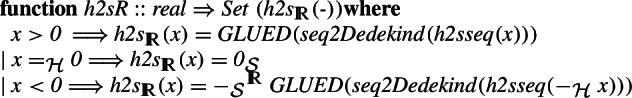


The two defined operations  and  are not as straightforward as for the naturals or rationals. We do nonetheless prove (details are only in the formalization) that they do indeed give an isomorphism and that this isomorphism preserves the basic arithmetic operations and the standard less than order. 
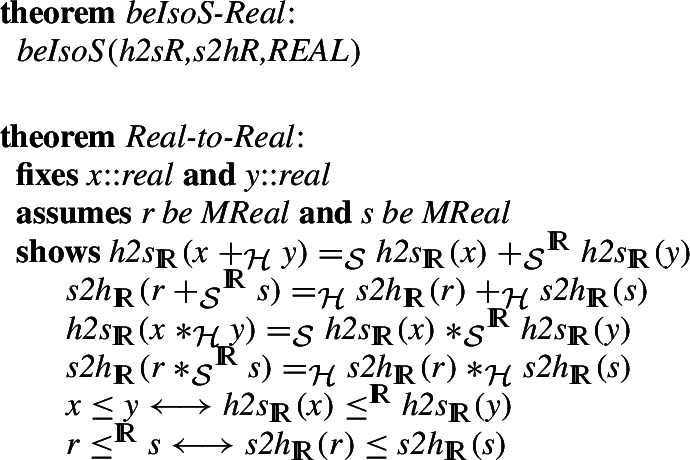


We are now ready to practically move proved theorems about numbers between HOL and Isabelle/Mizar.

## Algebra

The structure representations used in higher-order logic and set theory are usually different. This will be particularly visible when it comes to algebraic structures. In the Isabelle/HOL formalization, algebraic structures are type-classes while in set theory a common approach would be partial functions. We will illustrate the difference on the example of groups. A type $\alpha$ forms a group when we can indicate a binary function on this type that will serve as the group operation satisfying the group axioms. On the other hand, in the usual set-theoretic approach a group in set theory would consist of an explicitly given set (the carrier), and the group operation. With an intersection type system, the fact that the given set with an operation is a group is specified by intersecting the type of structures with the types that specify their individual properties (i.e., a group is a non-empty associative Group-like multMagma)

There are two more differences in the particular formalizations we consider, that we will not focus on, but we will only mention them in this paragraph and consider them only in the formalization. First, the existence and uniqueness of the neutral element can be either assumed in the group specification or derived from the axioms. We will not focus on that, as this is only the choice of a group axiomatization. Second, in the Mizar library, there are two theories of groups: additive groups and multiplicative groups. Rings and fields inherit the latter, while some group-theoretic results are derived only for the former. Even if the Isabelle/HOL group includes a field for the unit, we will ignore it in the morphism, since the set-theoretic definition does not use one. The neutral element along with the other properties is, however, necessary to justify that the result of the morphism is a group in the set-theoretic sense. 
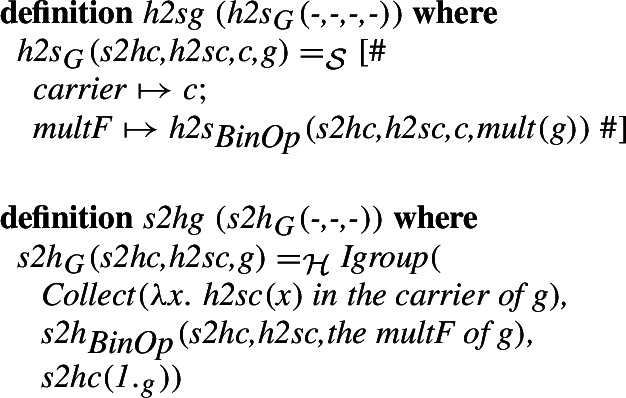


For the dual morphism, we indicate the result of the operation selecting the neutral element ( ) as the field needed in the construction of the type-class element. With its help, we can justify that the fields of the translated structure are translations of the fields. 
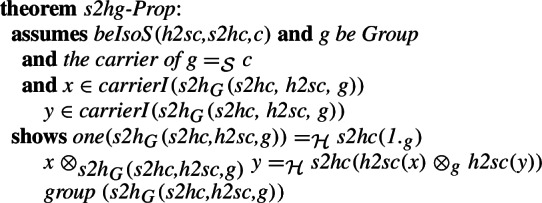


A number of proof assistant systems based both on higher-order logic (including Isabelle/HOL) and set theory (including Mizar) support inheritance between their algebraic structures. As part of our work aligning the libraries we also want to verify that such inheritance is supported in the combined library. For this, we align the ring structures present in the two libraries. The isomorphism between the structures is defined in a similar way to the one for groups, we refer the interested reader to our formalization.

We can show that the morphisms form an isomorphism and derive some basic preservation properties. The most basic one is the fact that the isomorphism preserves being a ring. 
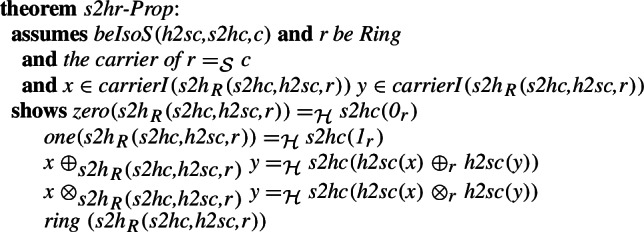


Finally, we introduce the equivalent of the definition of the integer ring introduced in the MML in [52]. We have previously discussed the semantics of Mizar structures and the way they are represented in Isabelle/Mizar in [27]. Here, with the previously defined isomorphisms for the subfields, we can show that  and  determine an isomorphism between the fields of the rings developed in Isabelle/HOL and the Mizar Mathematical Library. 
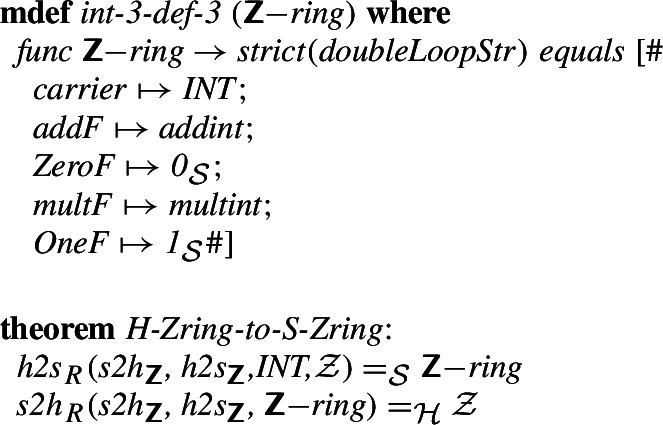


## Integrated Libraries: Practical Examples

We are now ready to use the existence of isomorphisms to automatically transform theorems about continuity of functions, including the Intermediate Value Theorem and the theorem that states that the image of a closed interval is a closed interval: 
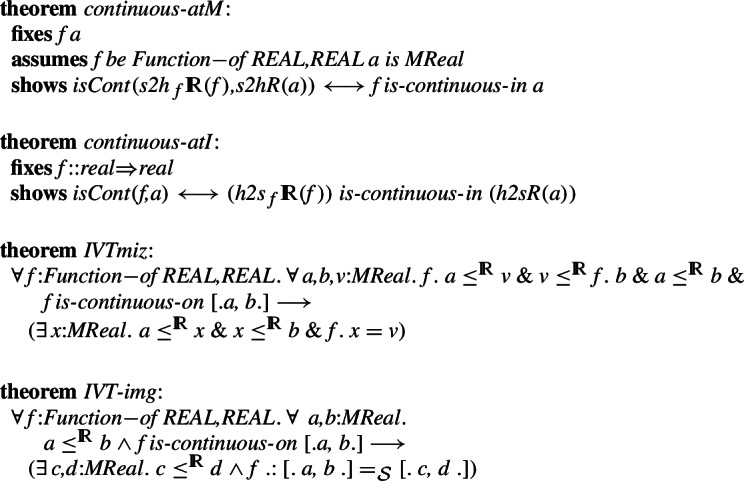


We also show the projection theorem, which again states that the homomorphisms agree and do not require any projections: 
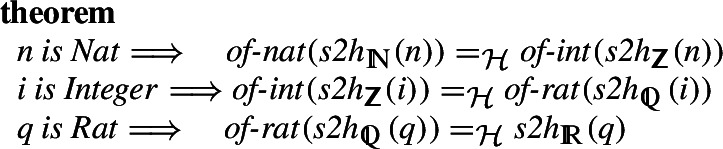


It is now possible to translate the Lagrange’s Four Squares theorem and Bertrand’s postulate between the libraries. We can prove the Isabelle/Mizar counterpart of the Isabelle/HOL theorem only using higher-order rewriting and the above properties. 
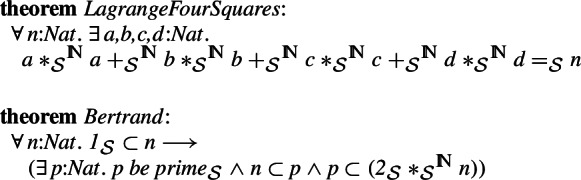


This allows translating the proved Fermat’s last theorem for powers divisible by 3 and 4 from Isabelle/HOL to Isabelle/Mizar. The original proof involved quite some computation and therefore has not been attempted in Mizar so far. However, thanks to the isomorphisms, the translated version can be proved automatically (higher-order rewriting combined with Isabelle/Mizar type automation): 
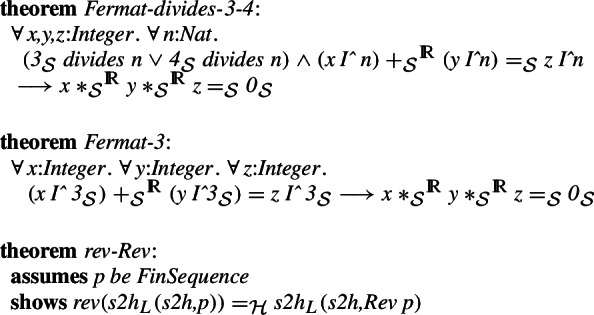


## Tarski’s Axiom vs. Grothendieck Universes

The theoretical part of our previous work [7] formally introduced a foundation for computer verified proofs based on higher-order Tarski–Grothendieck set theory (HOTG) and prove that this theory has a model if a 2-inaccessible cardinal exists. Referring to the former as the axioms of Tarski–Grothendieck is, however, slightly misleading, as there are two not immediately equivalent families of axioms. In particular, the two axiom families are equivalent assuming the axiom of choice. Additionally, the axiom of choice is a consequence of the Tarski axioms, but it is not the case for the Grothendieck formulation. Both of these facts are now also formalized in Isabelle, and shortly discussed in this section.

The formalization done in this section is done independently from Isabelle/HOL or Isabelle/Mizar as its goal is to formally justify that Tarski’s axiom A is valid in the model proposed in [7]. Recall, that Tarski’s axiom A is used in the Mizar library and in Isabelle/Mizar, whereas the existence of a Grothendieck universe is used for example in Egal.

Tarski’s Axiom A states that every set *N* is a member of some Tarski universe *M* which is closed under subsets, powersets, and every subset of the universe is either a member of the universe or is equipotent with that universe. To state this formally, the equipotence between the sets *X* and *Y* can be defined by a set of Kuratowski pairs, which defines a bijection from *X* to *Y* using only a minimal set of definitions, as it is done for example in the MML: 
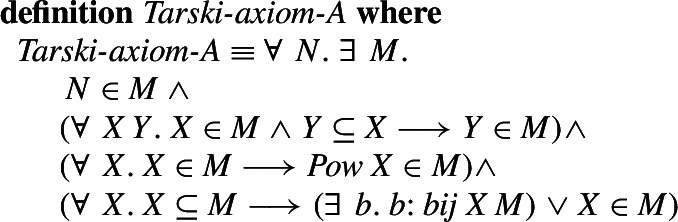
 In the Grothendieck approach, for an arbitrary set *X*, we can explicitly obtain the Grothendieck universe ${\textsf{Univ}}X$. The universe ${\textsf{Univ}}X$ is transitive (*Trans (Univ X)*), closed under union, powerset, and replacement (*ZFclosed (Univ X)*) and it is the smallest set (w.r.t. set inclusion) having these properties. 
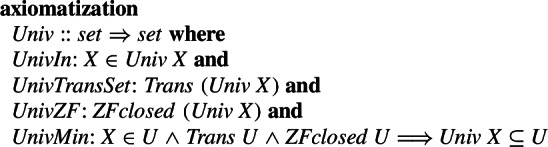
 To compare these two axiomatizations, we have previously shown in the higher-order logic of Egal that every Grothendieck universe, under the axiom of choice assumption, satisfies Tarski’s Axiom A (see [8]), but, not vice versa. Tarski universes, as opposed to Grothendieck universes, might not be transitive. We constructed such a Tarski universe of a set *N* that is a proper subset of ${\textsf{Univ}}N$ in [47] in the first-order logic of Mizar, as well as proved that ${\textsf{Univ}}N$ included in every Tarski universe of a set *N* if *N* is transitive.

In particular, using these properties, we proved in Isabelle that assuming HOTG and the axiom of choice, ${\textsf{Univ}}\,N$ is a Tarski universe, i.e., that in the model [7], Tarski’s Axiom A is valid. Rather than repeat the proofs already described in [8] we show the final statement that we proved under the axiom of choice as rendered by Isabelle: 
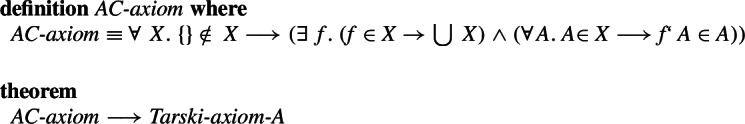


In order to even more closely show the adequacy of the HOTG model for importing the Isabelle/HOL proofs, one might also consider polymorphism, which is present in the foundations of the HOL families of provers. Andrew Pitts has provided a custom semantics to HOL that factors in polymorphism [50]. We however believe, that since the polymorphism in HOL is shallow (rank-one), it can be considered a notation for monomorphic HOL, namely all proofs can be translated to monomorphic ones and that the Grothendieck universes offer enough room for the quantification incurred by polymorphism. Extending the model to support all the custom extensions present in Isabelle/HOL (such as e.g., type classes [22] or local type definitions [30]) is left as future work.

## Related Work

Since proof assistants based on plain higher-order logic lack the full expressivity of set theory, the idea of adding set theory axioms on top of HOL has been tried multiple times. Gordon [17] discusses approaches to combine the power of HOL and set theory. Obua has proposed HOLZF [42], where Zermelo-Fraenkel axioms are added on top of Isabelle/HOL. With this, he was able to show results on partisan games, that would be hard to show in plain higher-order logic. Later, as part of the ProofPeer project [43], the combination of HOL with ZF became the basis for an LCF system, reducing the proofs in the higher-order logic part to a minimum (again, since there was no guarantee, that combining the results is safe). Kunčar [35] attempted to import the Tarski–Grothendieck-based library into HOL Light. Here, the set-theoretic concepts were immediately mapped to their HOL counterparts, but it soon came out that without adding the axioms of set theory the system was not strong enough. Brown [10] proposed the Egal system which again combines a specification of higher-order logic with the axioms of set theory. The system uses explicit universes, which is in fact the same presentation as given in this work. This work therefore also gives a model for the Egal system. Finally, we have specified [28] and imported [29] significant parts of the Mizar library into Isabelle. In this work, we only use the specification of Mizar in Isabelle and the re-formalized parts of the MML.

The idea to combine proof assistant libraries across different foundations also arose in the Flyspeck project [18] formalizing the proof of the Kepler conjecture [20]. Krauss and Schropp [33] specified and implemented a translation from Isabelle/HOL proof terms to set-theoretic proved theorems. The translation is sound and only relies on the Isabelle/ZF logic, however, it is too slow to be useful in practice, in fact, it is not possible to translate the basic Main library of Isabelle/HOL into set theory in reasonable time^2^ It is also possible to deep embed multiple libraries in a single meta-theory. Rabe [51] does this practically in the MMT framework deep embedding various proof assistant foundations and providing category-theoretic mappings between some foundations. Logical frameworks allow importing multiple libraries at the same time. In the Dedukti framework, Assaf and Cauderlier [1, 2] have combined properties originating from the Coq library and the HOL library. Both were imported in the same system, based on the $\lambda _\Pi$ calculus modulo, however, the two parts of the library relied on different rewrite rules.

Most implementations of set theory in logical frameworks could implicitly use some higher-order features of the framework, as this is already used for the definition of the object logic. The definition of the Zermelo-Fraenkel object logic [49] in Isabelle uses lambda abstractions and higher-order applications for example to specify the quantifiers. This is also the case in Isabelle/TLA [38]. These object logics are normally careful to restrict the use of higher-order features to a minimum, however, the system itself does not restrict this usage.

The first author together with Gauthier [15] has previously proposed heuristics for automatically finding alignments across proof assistant libraries. Such alignments, even without merging the libraries can be useful for conjecturing new properties [39] as well as improving proof assistant automation [14].

The fact that Grothendieck universes are the same as transitive Tarski classes has been formalized by Carneiro in Metamath.^3^

## Automated Transfer and Limitations of Current Work

In this section, we discuss transfer in higher-order logic based systems, transport in intuitionistic type theory, and the limitations of the current work when it comes to automating the transfer of theorems between the foundations.

Automating the transfer of theorems between different types in higher-order logic has a long history. Today, higher-order rewriting-based packages for the creation of quotient types are present in the libraries of most HOL-based proof assistants. These packages can automatically translate theorems from the raw types to the quotient types.

For example, HOL Light [19] includes the quot.ml package already since the nineties. This package defines two ML functions: lift_function and lift_theorem. The former automatically defines constants (often of higher-order function types) in a quotient type based on corresponding constants in a raw type. The latter ML function uses higher-order rewriting to transfer theorems that use the lifted constants to raw ones.

The procedure has been further improved by Homeier [23] in HOL4. The HOL4 quotient package allows an explicit declaration of properties of functions and relations (preserves and respects properties). These allow for quotients for polymorphic types. A similar architecture has been considered in the initial quotient package for Isabelle/HOL co-developed by the first author [34]. By further considering the interplay between the transfer in the outside and inside types it is possible to automatically quotient lists into finite sets with operations such as concatenation of a list of lists automatically translated into a finite set union.

The Isabelle/HOL quotient package has been modularized by Huffman and Kunčar [21]. The functionality has been separated into two packages: lifting and transfer. Lifting allows the automated translation of definitions in a source type to definitions in a target type (including quotient-based definitions). Transfer uses higher-order rewriting to move theorems between types. This modular construction allows the use of transfer also for cases of isomorphic types (including almost isomorphic ones, as was already the case for example with quotients), but where the target is actually not defined as a quotient of the source type.

A further improvement to the transfer mechanism in Isabelle/HOL has been developed by Kuncar and Popescu [30] in their work on local type definitions. There, the transfer package is extended to allow relativizing type-based statements to more set-based forms in a principled way.

In the context of intuitionistic type theory, translating theorems from types to their quotients is much more complex. This is because of the more intricate nature of equality in type theories, which in particular does not allow replacing equal things in all contexts (all above HOL packages rely not only on the axiom of choice but also on extensionality). An traditional approach to moving theorems between types that allows computation has been the use of setoids. This allows moving some theorems to quotients for example in the CoRN project [12].

More recently, foundations based on homotopy type theory [3] have been proposed. There, propositional equality between terms is interpreted as homotopy. The univalence axiom of Voevodsky [53] assumed in such foundations allows transporting properties and structures expressed over isomorphisms and equivalences. In its simplest variant, transport in HoTT/UF is an operation that takes a type family $P: A \rightarrow U$, a path $a = b$ in *A*, and returns a function $P a \rightarrow P b$ [40]. This allows transport between isomorphic types but does not take computation into account. This is further extended in cubical type theories [11]. There, it is possible to directly manipulate *n*-dimensional cubes based on an interpretation of dependent type theory in a cubical set model. Cubical type theories furthermore are specified in a way that allows Voevodsky’s axiom to be provable. Transport in cubical type theories [5] can take as input a line of types $A: I \rightarrow U$. This more primitive transport operation can however take computation into account. We are not aware of any automated tactics/packages allowing for transport of theorems between types in the same way as it is possible in Isabelle/HOL’s transfer package.

The work presented here, similar to the higher-order automated transfer packages, uses higher-order rewriting to translate the statements between the HOL types and the set-based representation, however, we have not been able to use the Isabelle transfer package for this. The reason for this is that on the Mizar side additional typing predicates are needed to express soft types and reasoning about these types is necessary. The Mizar soft types are additionally dependent. As such, we combine higher-order rewriting with our dedicated Isabelle/Mizar tactic for proving the Mizar type obligations (the mty tactic). As the tactic is responsible for Prolog-style type inference on the predicate level integrating its use with the existing Isabelle transfer package would be rather involved.

In principle, the equivalences provided by the isomorphisms allow translating the statements both in the assumptions and in the conclusions, however, we cannot directly use the transfer package, since type constraints not present on the term level in HOL correspond to explicit typing judgments in the set-theoretic types. Consider the isomorphism between the Mizar finite sequences and Isabelle/HOL lists. All the proved statements require the Mizar dependently typed assumptions stating that an argument is of a finite sequence type over some Mizar domain l be FinSequence-of t as well as an additional isomorphism for the domain. We have added the necessary assumptions to the theorems, and in the automated proofs, the Isabelle/Mizar type inference (including the automated proof of Mizar type inhabitation) is necessary to fulfill these obligations. We believe, that is it possible to augment the lifting and transfer packages to add soft type constraints on the term level and fulfill them wherever possible. The details are however unclear and are left as future work.

## Conclusion

We have used Isabelle HOTG to combine results proved in TG set theory with results proved in higher-order logic. This allows us to combine large parts of two major proof assistant libraries: the Mizar Mathematical library and the Isabelle/HOL library. Supplementary to the theorems and proofs coming from both, we define a number of isomorphisms that allow us to translate theorems proved in part of one of these libraries and use them in the corresponding part of the other library.

As part of the library merging, we have formally defined and proved in Isabelle the necessary concepts. Apart from porting proofs to Isabelle/Mizar, the isomorphism formalizations and the theorems moved using those amount to 10179 lines of proofs. The formalization is available at:

http://cl-informatik.uibk.ac.at/cek/ckkp-jar2022-hotg.tgz

Apart from higher-order and set-theoretic foundations, the third most commonly used foundation is dependent type theory. The most important future work direction would investigate combining the results proved here with those proved in such type-theoretic foundations.

So far, we have mostly moved results that have been proved in HOL to set theory. It could be also interesting to transfer the Brouwer’s theorem for *n*-dimensional case (the fixed point theorem [44], the topological invariance of degree, and the topological invariance of dimension [45]) that are essential to define and develop topological manifolds since the Mizar library results on manifolds are much developed than those in Isabelle/HOL [25].

## Footnotes

1 The Isabelle definitions and lemmas that directly correspond to the definitions and lemmas in the Mizar Mathematical Library have been names with the same identifiers in order to ease comparison. For example the Isabelle/Mizar definition *xboole-0-def-3* directly corresponds to the MML definition *XBOOLE_0:def_3* (colon is not allowed in Isabelle labels).

2 As part of an ongoing project to export Isabelle proof to Dedukti and the project exporting Isabelle to MMT [32] some of the proofs in Isabelle/Main are being currently optimized.

3 http://us.metamath.org/mpeuni/grutsk.html.
